# Wellbeing during a pandemic: An empirical research examining autonomy, work-family conflict and informational support among SME employees

**DOI:** 10.3389/fpsyg.2022.890265

**Published:** 2022-08-16

**Authors:** Najib Bou Zakhem, Panteha Farmanesh, Pouya Zargar, Abdulnasser Kassar

**Affiliations:** ^1^Department of Business Management, Girne American University, Kyrenia, Cyprus; ^2^Department of Information Technology and Operations Management, Lebanese American University, Beirut, Lebanon

**Keywords:** COVID-19, employees, SME, job autonomy, wellbeing, performance, work-family conflict, informational support

## Abstract

Individuals working in different industries were forced to change their work environments to their homes and quickly cope with technical and social changes not experienced before the occurrence of COVID-19 pandemic. This led to blurred boundaries between work and family roles, diminishing performance and wellbeing. Within the scope of the Research Topic “Workplace effects of COVID-19 on employees,” this research emphasizes on the positive impact of job autonomy provided by employers in reducing work-family conflicts. Moreover, the effect of work-family conflict on employees’ performance and wellbeing is analyzed. Furthermore, informational support is examined regarding its moderating effect to mitigate work-family conflicts and enhance wellbeing. A survey was administered among employees of small-medium enterprises in Lebanon, through purposive and convenience sampling with 198 participants. The data was analyzed using PLS-SEM, and the results show that job autonomy reduces work-family conflict. This in turn improves performance and wellbeing as individuals have more control on their tasks. Furthermore, informational support provided to the employees serves as a buffer between work-family conflict and wellbeing. These results can be beneficial for managers of small and medium enterprises, seeking to enhance the performance and wellbeing of their employees in the era of the pandemic. Similarly, scholars can benefit from theoretical premises of current study and the potential pathways for future analyses.

## Introduction

The outbreak of coronavirus pandemic (COVID-19) has upturned the lives of employees and the practices of organizations all over the world. Employees, who used to be physically present all or much of their time within the corporeal boundaries of their organizations while executing their jobs are expected to swiftly adapt to remote work environments. Organizations are also required to quickly cope with extreme disruptions and radical changes in the workplace and the social environment by navigating through an extraordinary epoch associated with technical, procedural, social, and physiological conditions. Productivity, satisfaction, and well-being are among the key factors hindered, and the increasing shift toward pandemic-induced telecommuting has blurred the boundaries between work and life commitments. In this sense, the current research addresses the impact of job autonomy on work-life conflict, and subsequently performance and wellbeing, following a string of studies focusing on this subject (Beauregard and Henry, 2009; Haar et al., 2014; Zheng et al., 2015) and particularly, amidst COVID-19 pandemic (e.g., Pluut and Wonders, 2020; Sheth, 2020; Cui and Li, 2021).

Due to lockdown and social distancing mandates, work-life boundaries are diminished as several activities and tasks are conducted at home such as working, socializing, and learning, shopping, and relaxing (Sheth, 2020). As organizations are seeking to cope with the changes imposed by COVID-19 through digital technologies which will most likely continue in the post-COVID 19 era (Fenwick et al., 2020), they have to be aware of disproportionate effects on employees, especially as teleworking has been viewed as unfamiliar and a radical move in work practices for many industries (Kramer and Kramer, 2020). Most notably, this transformation has started exacerbating work-family conflict (Novitasari et al., 2020) which is defined as an inter-role conflict that arises when job-related time and demands interfere with family-related time and duties (Netemeyer et al., 1996). Adjustment in organizational practices has become crucial for mitigating disruptions associated with telework and ensuring the continuity of business operations (Carillo et al., 2021). Specifically, organizations are required to provide employees with the necessary information, tools, independence, and autonomy to help them adapt to the “new normal” and balance between new or redefined forms of work requirements and family demands.

In this respect, this study poses a question regarding the extent to which job autonomy poses effects on work outcomes (i.e., work-family conflict) which in turn can influence professional and personal outcomes (i.e., performance and wellbeing) for SME employees. Moreover, this research addresses the gaps of literature in this context by including informational support as a moderating factor. The current study examines the impact of job autonomy on work-family conflict. Moreover, work-family conflict and its impact on performance of employees and their wellbeing is analyzed following recent findings in the extant literature (e.g., Novitasari et al., 2020; Pluut and Wonders, 2020; Sheth, 2020; Carillo et al., 2021). In addition, this research highlights the importance of organizations’ role through informational support as a moderator aiding employees to better face the work-induced conflicts. Hence, addressing the influence of job autonomy on employee performance and well-being in light of COVID-19 pandemic associated with work-family struggles resulting from telecommuting practices (Pulido-Martos et al., 2021).

Furthermore, previous research on telecommuting has concentrated on employees who selected this form of work by choice. However, the situation of COVID-19 is different and is the “new normal” in the post-pandemic era (Kniffin et al., 2021). Accordingly, the current research aims to contribute to the current understanding of job autonomy and work-family conflict and their interrelationship with both organizational and psychological outcomes (i.e., performance, and wellbeing). Additionally, this research furthers the geographical borders of the literature by providing empirical evidence from the Middle East region and particularly, Lebanon which is less examined by scholars. Subsequently, both scholars, and practitioners (e.g., SME managers) can benefit from the current findings to expand our knowledge on this subject, and to improve their organizational setting. In this respect, several questions are posed that are (a) to what extent job autonomy impacts work-family conflict of SME employees in Lebanon? (b) What are the effects of work-family conflicts on employee performance and employee wellbeing? and (c) How would informational support (i.e., information relating to latest health and safety developments, self-development opportunities for coping with role changes) provided by organizations improve employee wellbeing?

To address the aforementioned questions, this research focuses on important and challenging elements with complex natures. Psychological and professional impacts of a dire event such as the current pandemic, require extensive and thorough examination as it can echo for long after the pandemic. Accordingly, this research addresses noted gaps that are, (1) high levels of job autonomy, discretion, and self-responsibility granted by organizations to their employees during the pandemic help employees manage cognitive and emotional resources to alleviate work-family pressures and meet the contemporary work demands (Carnevale and Hatak, 2020); (2) the conjunction of newly-found work and family demands has an impact on employee performance (Carnevale and Hatak, 2020; Kniffin et al., 2021) and employee wellbeing (Cooke et al., 2020); and (3), informational support centered on healthcare updates and self-development aids employees to confront work-family conflicts and maintain wellbeing on satisfactory level (Cui and Li, 2021; Rasool et al., 2021). Understanding these effects can be beneficial for scholars to develop theoretical frameworks. Similarly, practitioners can benefit from such understandings and improve the functions of their organizations, particularly SMEs to increase performance, and enhance wellbeing of their employees (Rasool et al., 2020, 2021).

**FIGURE 1:**
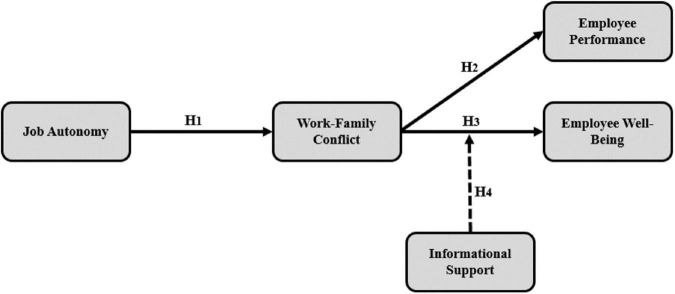
Theoretical model.

The research is structured in a manner that follows the setting and framework of the study by providing background and aims of the study in the introduction section, followed by theoretical background and literature review which forms the hypotheses of this research. Furthermore, theoretical model (Figure 1), sampling procedure, and measurements are provided to clarify research approach. Data analysis reports are provided alongside discussion upon findings that is followed by conclusion and implications. Lastly, limitations of the study and potential pathways for future scholars interested in the topic are noted.

## Theoretical background

### The impact of job autonomy on work-family conflict

Job autonomy is defined as the degree to which the worker has independence and flexibility in deciding how and when to conduct their job tasks (Breaugh, 1985). According to Thompson and Prottas (2006), the level of job autonomy acts in permitting or disabling family issues resulting from various work requirements. The constructive impact of job autonomy is justified by the higher amount of chances given to the employee to handle various stressful and difficult situations. In other words, this form of independence minimizes work-family conflicts by giving employees more control over their work and more ability to manage various family needs and organizational demands at the same time (Emre and De Spiegeleare, 2021). This research considers autonomy as a highly influential factor during times that employees are forced to work from home (i.e., telecommunication, real estate, education) and have their family roles combined with that of their careers.

Autonomy and discretion in executing work tasks allow the employee to have a wider job breadth and improve their ability in managing tasks. Ng and Feldman (2015) viewed job autonomy as a major factor playing a fundamental role in reducing conflicts resulting from the contradicting work-family demands and obligations. Other studies focused on other factors which would shape the relationship between job autonomy and work-family conflicts. For instance, Ahuja et al. (2007) found in a study targeting IT professionals who spend the majority of their time on client sites that job autonomy helps workers minimize work-family tensions. Similarly, a study recently conducted by De Clercq and Brieger (2021) on women entrepreneurs shows that independence in conducting tasks is considered a source of energy for women to maintain equilibrium between their business duties and family roles. While gender difference is not looked into in the current study, overall wellbeing and performance especially related to work-family conflicts are emphasized. Many SMEs did not have the necessary equipment, systems, or strategies to handle the pandemic and its speed of posed changes.

The social-cognitive theory provides a solid framework for explaining the role of job autonomy in reducing work-family conflicts and improving task efficiency. According to Bandura (2002), one aspect of the social-cognitive theory is that people practice control in order to recognize the rewards or benefits. SME employees often have a set of tasks that are followed routinely and therefore a certain level of autonomy exists. However, when these roles are mixed with remote work situations, the control that is perceived over job is influenced. Furthermore, Bandura (2002) assumes that personal control is a goal-directed manner. This point of view has a substantial implication for understanding autonomy and fostering balance between work duties and family responsibilities.

More specifically, by projecting the social-cognitive theory on job autonomy, it is suggested that individuals perceiving major benefits resulting from work independence will understand the subsequent utility and be motivated toward work. For instance, an individual who views productivity as a favorable outcome will likely view autonomy as an expedite opportunity for attaining higher performance levels (Langfred and Moye, 2004). Other performance-related benefits which justify job autonomy lowering the impact of work-family conflicts on performance include interest, ingenuity, flexibility, and faster learning (Deci and Ryan, 1987). In the light of what was noted and considering the context of current research, we assume that job autonomy aids employees in alleviating work-family conflict and meeting the contemporary work demands during the pandemic, particularly for the case of SME employees. Accordingly, the following hypothesis is formed:

**H_1_:** *Job autonomy can reduce work-family conflicts.*

### Work-family conflict and employee performance

Work-family programs have significant economic effects pertaining to cost effectiveness, productivity, and attraction of labor force (Clifton and Shepard, 2004). By contrast, lack of work-family facilitation has an adverse effect on performance outcomes (Van Steenbergen and Ellemers, 2009). From an organizational viewpoint, one notable idea of examining work-family interfaces is that workers become capable of managing work-family realms and keeping flawless boundaries between these two dimensions, leading to higher performance in work (Odle-Dusseau et al., 2012; Wang et al., 2019). A cross-sectional study examining the impact of work-family conflict and work-family facilitation practices on employee performance has shown that such experiences strongly sway the employees’ managerial competencies (Graves et al., 2007).

Lower performance has also been reported among non-managers related to work-family conflict (Witt and Carlson, 2006). Moreover, in a study addressing school teachers, Hakanen et al. (2006) considered fruitless job performance and poor job embeddedness are classified as rational consequences of emotional strain resulting from conflicting work-family roles among teachers. Recently, Liao et al. (2019) stated that work-family conflict has an unfavorable impact on career outcomes. This study reconciles with the study conducted by Xian et al. (2021) who found work-family struggles significantly weaken job satisfaction and productivity, especially among parents with a single child.

This study focuses on the impacts of global pandemic on performance as it is a highly important factor in the same line as wellbeing. Work-family policies established by the human resources department is strongly linked to the attainment of high levels of organizational performance (Perry-Smith and Blum, 2000; Cui and Li, 2021). Another study investigating the efficiency of work-family balance programs has shown that flexibility practices have a positive impact on employee performance in terms of number of sales (Konrad and Mangel, 2000). Furthermore, the recent study conducted by Pulido-Martos et al. (2021) shows that if the pandemic-induced telecommuting imposed on employees is supported by conditions similar to the face-to-face setting, this would help organizations mitigate unfavorable results that are caused by distant working.

The “Role Theory” outlines the foundation for explaining work-family conflicts and their consequences on employee performance by proposing that each role is associated with an established set of obligations, rights, potentials, and actions that an individual is expected to face (Biddle, 1986). According to the role theory, as work-family conflicts aggravate, individuals’ powers are dispersed. Particularly, this theory indicates that role overburden and role conflict are deemed inimical to work performance (Carlson et al., 2019). This means that employees suffering stress resulting from unclear job roles will be more likely disgruntled with their job and less motivated to accomplish their tasks. Employees will no longer be able to efficaciously engage in work, due to the inadequate amount of resources and power available within the work sphere (Rizzo et al., 1970). In this regard, the depletion of work resources will compel employees to exert more efforts to meet job demands and their attitudes toward their job and the organization as a whole can become negative (Cui and Li, 2021). SME employees in the current context face conflicts between life domains that are caused by the pandemic and the subsequent work setting that is stressful and can impact engagement and motivation. This is linked to an abrupt and forced change in the environment of work such as teachers (Telyani et al., 2021).

Although there is relatively enough evidence that work-family conflicts are adversely linked to employee performance, literature is still scarce regarding the impact of the newly emerged work-family conflicts resulting from the blurred boundaries during the COVID-19 pandemic. Besides, telecommuting has been viewed as a far-reaching move in work practices for many of the employees who used to execute their jobs within the physical realm of their organizations in the pre-COVID 19 period (Kramer and Kramer, 2020). Hence, in compliance with the Research Topic and recent findings (e.g., Carnevale and Hatak, 2020; Kniffin et al., 2021) concerning the need for empirical studies which would tackle the impact of pandemic on employees, we assume the following hypothesis with regards to SME employees.

**H_2_:** *Work-family conflict negatively affects employee performance.*

### Work-family conflict and employee wellbeing

Wellbeing is broadly defined as the state through which individuals maintain mental, emotional, and physical health in regard to work and non-work experiences (Nielsen et al., 2017). Wellbeing of employees is highly affected by the amount of happiness or irritation resulting from the job itself or from the individual’s personal interactions with family members, colleagues, supervisors, subordinates, and friends. Danna and Griffin (1999) accentuate that wellbeing encompasses both psychological considerations (e.g., lack of anxiety, sorrow, and emotional exhaustion) and biological considerations (e.g., absence of fatigue). Similarly, Cartwright and Pappas (2008) view hedonistic and satisfaction levels as indicators of psychological wellbeing.

Employee wellbeing is strongly shaped by organizational tradeoffs including job design, organizational structure, safety and compensation packages (Carnevale and Hatak, 2020) as well as personal needs going beyond the occupational realm (Luthans et al., 2013). A sense of control for these various requirements is desirable to maintain well-being. This sense is reflected through organizational work-life balance programs, which aim at fulfilling employees’ needs for various work and non-work roles (Obrenovic et al., 2020). For the case of current research, the aforementioned notion is used to highlight the importance of conflict between life domains and wellbeing of individuals working in SMEs. In this respect, this study focuses on results that can foster wellbeing as a core concept.

Work-life conflict lowers wellbeing and leads to higher levels of stress (Fotiadis et al., 2019; Babic and Hansez, 2021; Telyani et al., 2021). More specifically, tension in work-family relations decreases wellbeing by exhausting intellectual resources and increasing psychological burden (Voydanoff, 2002; Eby et al., 2005). Likewise, Fiksenbaum (2014) accentuates that employees encountering work-family conflicts report fewer levels of life satisfaction and less energy available to invest in their jobs. This is consistent with the study conducted on parenting in view of COVID-19 by Goldberg et al. (2021) who assure that the increasing work-family obligations resulting from the pandemic have resulted in increasing amount of anxiety, vulnerability, and strain among working parents.

The “Conservation of Resources” theory (COR) lays off the theoretical framework for explaining the dynamics within employees’ resources pool and their influence on wellbeing and threats hampering it (Hobfoll et al., 2018). The COR theory elucidates how work-family conflicts result in negative consequences on employees’ psychological wellbeing (McNall et al., 2010; Samma et al., 2020), explaining how a positive workplace behaviors are less likely to appear when work-family conflict exists. This theory is based on the belief that humans are interested in conserving the current resources and obtaining new ones (Halbesleben et al., 2014). According to Hobfoll (2002), work and family constitute a pool of resources where any prospective threat, advancement, or loss in one of these aspects unquestionably affects the other. This implies that utilizing too much resources into work generates family-related problems and vice versa, resulting in work-family stress and occupational burnout.

The increasing demands in any of these domains would weaken individuals’ energy and resources available for the other and require commitment of both physical and emotional resources to overcome the issue (Hobfoll, 2002; Samma et al., 2020; Zhou et al., 2020). Under the realm of blurring work-family roles, available resources would be misplaced, thus initiating an unfavorable or a negative conflict-being status. In the study of Grant-Vallone and Ensher (2001) addressing expatriates in Switzerland, work-personal life tension was strongly linked to employee depression and was classified as a major concern for their health and wellbeing. A recent study similar to the context of this research conducted in China looked into different causes of work-family conflict, where personal characteristics (e.g., emotional intelligence) and organizational aspects (e.g., workplace anxiety) were found significant (Cui and Li, 2021).

Work-family boundaries became highly indecipherable due to the changes in family role, technological advancements, and the increased employer expectations of the employee’s ability to work at anytime from anywhere. For this reason, employees are suffering high levels of emotional strain and confronting issues relating to psychological wellbeing (Obrenovic et al., 2020). Considering how employees of SMEs had to shift their work to a new environment where family roles are made bold, and as per Carnevale and Hatak (2020) and Cooke et al. (2020) who argue that the coincidence of work and family demands during the COVID-19 era might have a substantial impact on employee wellbeing, while referring to COR theory, the following hypothesis has emerged:

**H_3_:** *Work-family conflict negatively affects employee well-being.*

### The moderating role of informational support

The availability of information relating to the latest updates in the healthcare context and self-development opportunities during the coronavirus pandemic would help employees mitigate work-family conflicts and improve wellbeing (Carnevale and Hatak, 2020). The role of organizational support on various work-related factors has been noted as a significant matter in the extant literature (Rasool et al., 2021). Kopp et al. (2011) argue that human resource professionals have to listen to the employees’ concerns, support them, and bring their voice into the decision-making process during crisis. Employees have to be informed about the latest information relating to business state and work updates as well as having emotional, social and instrumental support (Kim et al., 2007; Rasool et al., 2021). Eisenberger et al. (2002) reports that supervisory support is crucial for helping employees overcome the negative consequences of crises. Specifically related to pandemic context, Rapaccini et al. (2020) state that firms should exhibit a degree of preparedness by sharing the necessary information with their employees needed for digitalizing the work processes to help employees cope with the situation and improve their work capabilities with high level of confidence.

Wooten and James (2008) accentuate that crisis communication is crucial for ensuring organizational continuity and employee wellbeing during unfavorable circumstances. Crisis communication involves sharing truthful and continuous information relating to the business state, taking different perspectives into account, hearing constantly from employees, and ensuring employee wellbeing is maintained. It also requires leaders to lavish their emotional acuity to respond empathetically to employee needs and concerns during crisis. In addition, Ross et al. (2017) confirm that technologically-driven communications help employees reduce work-family conflicts by providing employees with high level of flexibility and control. Similarly, Bove and Benoit (2020) proclaim that support is the key for overcoming the critical period during COVID-19 pandemic. Such support involves implementing safety signals, building strong communications, and providing employees with necessary resources needed to work remotely.

As support (emotional and informational) significantly reduces role demands employees and their capabilities for handling tasks during crises, it exhibits a moderating influence on work outcomes (i.e., wellbeing and reduced work-family conflict) (e.g., Greenhaus et al., 2006; Neto et al., 2016). A similar effect is reported regarding moderating effect of informational support on work-family conflict and other work or personal outcomes (e.g., burnout, and life satisfaction), further influencing employees’ wellbeing (Lingard and Francis, 2006; Goh et al., 2015). This has been reported in the literature that due to reduced demands in work domain, work-family conflicts can be managed more effectively and thus, individuals can have a better level of wellbeing (Achour et al., 2017).

For the purpose of minimizing physical and emotional stress, leaders are expected to provide information which primarily shows high level of concern to their happiness and wellbeing (physical and psychological) (Tuzovic and Kabadayi, 2020). Molino et al. (2020) report the importance of HRM techno-communication and intervention for fortifying employees, helping reduce work-family stressors, and nurturing positive implementation of remote work strategies. Notably, implementation of the telecommuting practices imposed by the pandemic varies from one organization to another depending on the availability of resources and the level of preparedness of its employees (Meyer et al., 2021; Raghavan et al., 2021). A consensus is observed among scholars related to the moderating impact of informational support on wellbeing and work-family conflict in various cases and different settings (e.g., Greenhaus et al., 2006; Goh et al., 2015; Neto et al., 2016; Achour et al., 2017; French et al., 2018; Anand and Vohra, 2022), the assumption is made upon updating employees working for SMEs with the information needed to take the necessary health measures and cope with the rapid changes in the work processes to minimize the negative influence of work-family conflicts on their physical and psychological wellbeing.

**H_4_:** *Informational support moderates the relationship between work-family conflict and wellbeing among SME employees.*

## Research design

### Sampling and data collection

This study takes a quantitative approach through a purposive and convenience sampling method. For calculating the required sample size, G*power software was deployed with statistical power of 80% and effect size of 0.01 (Faul et al., 2007), which was combined with suggestions of Hair et al. (2017) (Min R^2^ = 0.10, α = 0.01). The resulting range was calculated between 131 and 180. Hence, a sample that is more than 180 is deemed appropriate for current analyses to draw significant results from the data. Through an online survey, quantitative cross-sectional data was gathered from employees working for SMEs in Lebanon, who were forced to change their work modality due to COVID-19 pandemic.

Purposively, the sample was selected from the staff, who strictly worked in physical offices. This included employees from real estate, insurance, financial and education sectors. Moreover, all respondents had a minimum of one child and 40–60 working hours per week. Managers were informed and objectives of the research were established in a thorough manner. Using this criteria, a pilot test was conducted with a sample of 30 employees from a selected SME which was not included in the final analysis. The pilot is attested for extent of validity, reliability and understandability of the items. Through a convenience sampling method for gathering data from 10 unique SMEs, a total of 250 questionnaires were distributed, from which employees returned 211 (84.4% response rate). After screening the data, 13 surveys were omitted due to bias and response error (e.g., incomplete answers).

Respondents’ profile included 53% males and 47% females with average age being 35.2 (SD = 5.5). Notably, data confidentiality was assured to the respondents; proximal separation was used (to provide information regarding daily activities). Collinearity was tested with VIF less than 3.3 which eliminated the concerns regarding common method bias (Podsakoff et al., 2003; Kock, 2015; Jordan and Troth, 2020). Participation was completely voluntary and original data did not include any personal and/or sensitive information. Both gender and age were controlled as exogenous factors.

### Measurements

Job autonomy was measured using the scale developed by Morgeson and Humphrey (2006) in which three dimensions are described as work-scheduling, decision-making, and work methods autonomies. Furthermore, work-family conflict items were selected from the work of Frone (2000) with two dimensions that are namely, work-to-family and family-to-work interference. Informational support was derived from Madjar’s (2008) while employee wellbeing and performance were measured based on the scales developed by Arnold et al. (2007) and Singh (2019) respectively. All questions were measured with a 5-item Likert scale ranging from strongly agree (1) to strongly disagree (5). Measurements were examined regarding reliability and validity and the reports are shown in Table 1.

**TABLE 1:** Measurement model.

Constructs	Sub-dimensions	Indicators	Outer Loadings	Alpha	Rho A	CR	AVE
Job Autonomy	Work Scheduling	WS1	0.730	0.813	0.822	0.811	0.641
		WS2	0.832				
		WS3	0.921				
		WS4	0.702				
	Decision Making	DM1	0.824	0.762	0.820	0.761	0.735
		DM2	0.841				
		DM3	0.845				
		DM4	0.956				
	Work Methods	WM1	0.867	0.728	0.744	0.728	0.717
		WM2	0.772				
		WM3	0.809				
		WM4	0.982				
Employee Wellbeing	—	WB1	0.843	0.873	0.842	0.866	0.732
		WB2	0.827				
		WB3	0.874				
		WB4	0.746				
		WB5	0.789				
		WB6	0.898				
		WB7	0.709				
Informational Support	—	ES1	0.886	0.890	0.938	0.856	0.575
		ES 2	0.808				
		ES3	0.803				
		ES4	0.723				
Employee Performance		EP1	0.850	0.806	0.911	0.845	0.582
		EP2	0.841				
		EP3	0.823				
		EP4	0.732				
		EP5	0.744				
Work-Family Conflict	Work-to-family Interference	WFI1	0.823	0.802	0.830	0.841	0.721
		WFI2	0.845				
		WFI3	0.757				
	Family-to-work Interference	FWI1	0.887	0.771	0.757	0.720	0.678
		FWI2	0.890				
		FWI3	0.703				

### Analysis

To analyze the proposed model of this study, PLS-SEM was used as latent variables exist in the model, normal distribution was of no concern, and statistical significance can be achieved with smaller sample size (Hair et al., 2017).

## Results

Outer loadings of the measurement model were found to be above 0.7, while Rho A, alpha and composite reliability met the satisfactory threshold of above 0.7 and below 0.9 (Jöreskog, 1971; Diamantopoulos et al., 2012; Dijkstra and Henseler, 2015; Hair et al., 2019). Moreover, average variance extracted is above 0.5, exhibiting sufficient convergent validity alongside heterotrait-monotrait (HTMT) value that is below 0.85 (Henseler et al., 2015; Hair et al., 2017). These values are shown in Tables 1, 2, stating that the measurement model is qualified.

**TABLE 2:** Heterotrait-Monotrait ratio (HTMT).

	JA	WFC	IS	EP
JA				
WFC	0.712			
IS	0.602	0.630		
EP	0.702	0.608	0.741	
EWB	0.681	0.803	0.580	0.552

In addition to validation of the measurement model in Tables 1, 2, the structural model is analyzed and similarly confirmed. In this respect, indices such as, normal fit (NFI = 0.922), standardized root mean square residual (SRMR = 0.023), and VIF were found below 3 which imply no issue regarding multicollinearity alongside both R-square and Q-square implying empirically sound results for in-sample predictive power and relevance (Henseler et al., 2009, 2014; Hair et al., 2019). These results present a “fit” statistical model shown in Table 3.

**TABLE 3:** Structural model assessment and hypothesis testing.

Effects	Relations	β	t-statistics	F^2^	Decision
**Direct**					
H1	JA → WFC	−0.301	−5.203***	0.113	Supported
H2	WFC → EP	−0.413	−6.836***	0.154	Supported
H3	WFC → EWB	−0.207	−2.969**	0.087	Supported
**Interaction**					
H4	WFC*IS → EWB	−0.355	−1.233*	0.116	Supported
**Control Variables**					
	Gender → EWB	0.143	2.466*		
	Age → EWB	0.107	2.054*		

R^2^_WFC_ = 0.39/Q^2^_WFC_ = 0.19.

R^2^_EP_ = 0.48/Q^2^_EP_ = 0.27.

R^2^_EWB_ = 0.63/Q^2^_EWB_ = 0.38.

SRMR: 0.023; NFI: 0.922.

* 0.05, ** 0.01 and *** 0.001.

## Discussion

In accord with the aim of this study to investigate the effect of job autonomy on WFC, *hypothesis 1* was supported which shows consensus with the existing literature of the subject Emre and De Spiegeleare, 2021; De Clercq and Brieger, 2021) while developing its implications in the Middle East and particularly, Lebanon. This falls within social-cognitive theory and its premises as SME staff need a certain level of autonomy to conduct their daily tasks. As remote work can hinder other aspects of life, job autonomy can be a significant matter for controlling conflicts between work and family domains.

This is further linked to the negative effect of WFC on performance of employees as stated in *hypothesis 2*. Current findings show consensus with the literature regarding the aforementioned effects (e.g., Carlson et al., 2019; Rasool et al., 2020), implying that WFC depletes individuals from their resources, leading to lowered performance. Decreased performance can be revealed in forms of less motivation, disengagement, and stress in an increasing level as job demands increase. Embedded in the premises of role theory, our results show that performance can be significantly hindered for SME employees through WFC. This can lead to dire outcomes for the firm as individuals can have their attitudes toward the firm changed in a negative manner (Cui and Li, 2021).

Similarly, *hypothesis 3* was supported, which states the negative effect of WFC on employees’ wellbeing within the context of SMEs in Lebanon that is the focus of current research. Notably, various scholars have found similar results while examining different cases such as, Pakistani SEMs (Samma et al., 2020), which implies that wellbeing can be jeopardized during remote work. With regards to COR theory, psychological wellbeing of staff can be negatively affected *via* WFC, which can hinder positive behavioral outcomes in the workplace (Hobfoll et al., 2018; Carnevale and Hatak, 2020; Cooke et al., 2020; Obrenovic et al., 2020; Samma et al., 2020; Zhou et al., 2020; Wang et al., 2022).

Current findings also suggest that the relationship between WFC and wellbeing of employees can be greatly influenced by informational support, leading to acceptance of *hypothesis 4*. Linked to the theoretical framework of the study, this funding suggests that employees can have reduced demands in their work due to provided information pertaining various aspects of the business (i.e., changes in tasks, requirements, health-related issues, usage of new systems and other strategies). Equipping staff with necessary information regarding processes of the company with the aim of emotional, instrumental, and informational aid can act as a protector when WFC exists, especially during remote work. Moderating effect of informational support among SME staff can enhance their wellbeing as found in the literature in China (Rasool et al., 2021) and various other cases (e.g., Goh et al., 2015; Neto et al., 2016; Achour et al., 2017; Anand and Vohra, 2022).

## Conclusion and implications

The current findings are in consensus with the extant literature and recent findings while contributing to our understanding regarding wellbeing of employees during Covid-19 pandemic. As this study is conducted within the Research Topic “Workplace effects of Covid-19 on employees” for Frontiers in Psychology, the theories that are used in this research are used to derive tangible results. In doing so, social cognitive theory is embedded as job autonomy is shown to have a negative impact on SME employees’ work-family conflict (Table 3). This is while both performance and wellbeing were jeopardized by existence of conflict in the lives of individuals, leading to negative outcomes. We emphasize son the vitality of psychological impacts that hindered wellbeing can have on long-term health of individuals, especially in the long-term as it can impact the future of SMEs in the post-pandemic era. This further becomes more vivid when factors such as, stress, anxiety, efficiency, rights and other negative elements (e.g., lack of social interactions) are combined in a setting such as the pandemic. These findings are further linked to role theory, which addresses the aforementioned elements with regards to expectations (Biddle, 1986; Carnevale and Hatak, 2020; Cui and Li, 2021; Telyani et al., 2021).

Furthermore, current findings suggest that informational support carries a moderating effect, in a way that the negative effect of work-family conflict on wellbeing is reduced. This is of significant importance as individuals, who work in SMEs had to forcefully combine different life domains, leading to points of conflict between work and family. Linked to conservation of resources theory, finite resources used under pressure of work and family can have dire impacts on individuals’ wellbeing, especially in psychological aspects (Hobfoll et al., 2018; Carnevale and Hatak, 2020; Cui and Li, 2021; Landolfi et al., 2021). Obtaining new resources (i.e., informational support) is a crucial matter for individuals as they can then distribute it among conflicting domains.

These findings provide empirical evidence regarding COR and its implications during Covid-19 pandemic for SME personnel and their wellbeing. We emphasize on the role of managers and organizational systems (i.e., HRM) to provide necessary tools and equipment combined with the information that enables a smooth path for adaptation to change. In the era after the pandemic, and for the future of SMEs, it is essential that mangers implement effective strategies to create a work environment resilient enough to provide necessary support to their staff. This becomes more vivid in the future and for the next pandemic or other crises.

When individuals are equipped with the knowledge that is required for uncertain times such as the pandemic, stress, anxiety, time consumption, and other negative factors can be better handled. This, combined with job autonomy, can have vivid effects on performance and wellbeing of employees among SMEs. Due to relatively small number of employees, the managers of these firms can implement strategies, in which wellbeing is focused and therefore, initiatives are deployed that provide adequate and sufficient information of company policies, actions, and leadership. This can greatly impact wellbeing of individuals during the pandemic while work environments remain in an uncertain state.

## Limitations and recommendations

There are several factors that have limited the conduct process of this research. Notably, the current model can be analyzed with relevant factors such as, personal characteristics or behavior (e.g., citizenship or proactive behavior), leadership, and coping mechanisms. Future studies can further develop the theoretical and practical implications of current research through longitudinal data, examining variations in time and the changes and effects in the future of SMEs. This can provide a more thorough understanding on the effectiveness of implemented strategies. In-depth understanding of such complex matters can be gained through qualitative interviews with decision-makers and/or managers to better understand the challenges for maintaining resilience and adapting to changes caused by the pandemic.

## Data availability statement

The original contributions presented in this study are included in the article/supplementary material, further inquiries can be directed to the corresponding author.

## Ethics statement

The studies involving human participants were reviewed and approved by GAU Ethical Committee—PF (Head of Ethics Committee). The patients/participants provided their written informed consent to participate in this study.

## Author contributions

NZ: writing and data collection. PF: supervision. PZ: analysis and final writing. AK: proofreading and advising. All authors contributed to the article and approved the submitted version.
